# Elucidating the mechanism of corneal epithelial cell repair: unraveling the impact of growth factors

**DOI:** 10.3389/fmed.2024.1384500

**Published:** 2024-04-04

**Authors:** Jinjin Gong, Gang Ding, Zhongkai Hao, Yuchun Li, Aijun Deng, Chenming Zhang

**Affiliations:** ^1^School of Clinical Medicine, Shandong Second Medical University, Weifang, China; ^2^Department of Ophthalmology, Jinan Second People’s Hospital, Jinan, China; ^3^Wuxi No. 2 Chinese Medicine Hospital, Wuxi, China

**Keywords:** cornea, corneal epithelial cells, growth factors, growth factor receptor, repair mechanism

## Abstract

The repair mechanism for corneal epithelial cell injuries encompasses migration, proliferation, and differentiation of corneal epithelial cells, and extracellular matrix remodeling of the stromal structural integrity. Furthermore, it involves the consequential impact of corneal limbal stem cells (LSCs). In recent years, as our comprehension of the mediating mechanisms underlying corneal epithelial injury repair has advanced, it has become increasingly apparent that growth factors play a pivotal role in this intricate process. These growth factors actively contribute to the restoration of corneal epithelial injuries by orchestrating responses and facilitating specific interactions at targeted sites. This article systematically summarizes the role of growth factors in corneal epithelial cell injury repair by searching relevant literature in recent years, and explores the limitations of current literature search, providing a certain scientific basis for subsequent basic research and clinical applications.

## 1 Introduction

**Figure 1:**
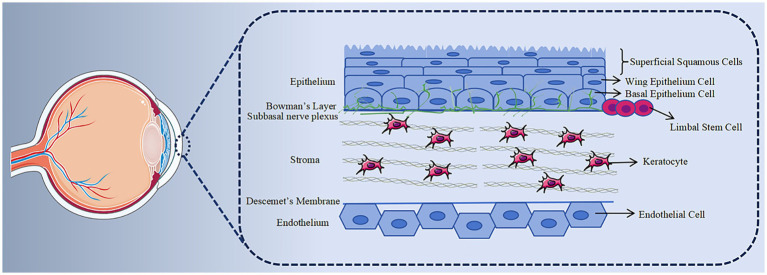
Histology of cornea. The cornea is structurally divided into five layers. The anterior epithelial layer comprises 5–7 layers of renewable epithelial cells. Behind the epithelial basement membrane lies the Bowman membrane, consisting of collagen fibers. The thickest layer, the stroma, primarily consists of keratocytes and collagen fibers, crucial for maintaining corneal transparency. Descemet, generated by the endothelium, is a transparent, elastic thin film with no distinct structure but possesses strong resistance. The endothelium is formed by a layer of hexagonal endothelial cells, incapable of regeneration.

The cornea, in direct contact with the external environment, stands out as one of the body’s tissues with the most dense innervation (1). Its structure is crucial for preserving the health and functionality of the ocular surface (Figure 1 shows the anatomical structure of the cornea). Damage to the corneal epithelium can result in corneal infections, ulcers, scar formation and vision loss ultimately. In recent years, factors such as surgical trauma, drug use, and infection have contributed to a rising number of patients with corneal epithelial injuries. As a result, the repair of corneal epithelial injuries has emerged not only as a prominent topic in basic scientific research but also as an urgent clinical problem that requires attention.

The corneal epithelium plays a crucial role in safeguarding the eye’s barrier, stabilizing the tear film and maintaining the microenvironment of the ocular surface (2). Currently, the model governing corneal epithelial homeostasis relies on the XYZ hypothesis. According to this framework, the migration of limbal stem cells (LSCs) towards the central region of the cornea (X), combined with the vertical proliferation and differentiation of basal cells (Y), balances with the shedding of squamous cells (Z) from the epithelial surface. Healthy eyes are continuously bathed in tears containing growth factors, essential substances for maintaining the normal function of ocular surface tissue (3). Due to the unique anatomical location of the cornea, it is particularly susceptible to various injuries (4). When damaged, there is an upregulation of growth factors in tears, which target the cornea through relevant signaling pathways, thereby promoting corneal epithelial repair (5) and maintaining corneal epithelial homeostasis. Therefore, growth factors play a pivotal role in repairing corneal epithelial injuries and maintaining the normal microenvironment of the corneal epithelium.

In consideration of the aforementioned, this review aims to elucidate the roles played by various growth factors in the repair of corneal epithelial cells and comprehensively analyze their respective mechanisms of action. The overarching objective is to establish a robust scientific foundation that can serve as a springboard for subsequent basic research endeavors and clinical applications. The ensuing discussion will delve into the intricate interplay of growth factors within the context of repair mechanisms.

## 2 Growth factors involved In repair mechanisms

### 2.1 Epidermal growth factor

Epidermal growth factor (EGF) stands as one of the earliest identified single-chain peptides recognized for its ability to stimulate cell growth, playing a pivotal role in wound healing and maintaining tissue homeostasis by regulating cell survival, growth, motility and differentiation (6). In instances of corneal epithelial cell damage resulting from trauma, surgery or infection, EGF facilitates the migration and proliferation of corneal epithelial cells through the activation of its receptor EGFR/ErbB and subsequent binding. Consequently, this process promotes the effective repair of corneal epithelial injuries (7, 8). During the initial phases of corneal epithelial damage healing, EGFR1/ErbB1 tyrosine kinase instigates cellular signaling, activating downstream effectors such as the type III phosphoinositide 3-kinase (PI3K)—protein kinase B (Akt) axis and extracellular signal-regulated kinase (ERK). This orchestrated activation contributes significantly to the overall repair mechanism of corneal epithelial injuries (9). Furthermore, substance P also emerges as a noteworthy contributor to corneal epithelial injury repair, operating through the activation of EGFR and downstream signaling molecules, such as Akt (10). Abnormal activation of the EGFR-PI3K-AKT and ERK signaling pathways may result in increased cell apoptosis, decreased cell proliferation and delayed wound closure (11). The EGFR signaling pathway can further activate nuclear factor kappa-B (NF-κB) and histone deacetylase 6 (HDAC6). NF-κB, in turn, activates the transcription inhibitor CCCTC binding factor (CTCF) while downregulating the paired box gene 6 (PAX6), mediating the migration and proliferation of corneal epithelial cells. Simultaneously, HDAC6 promotes the migration of corneal epithelial cells and contributes to injury repair (12, 13).

There are four EGF receptors, with EGFR1 showing relatively high expression in corneal epithelial cells and demonstrating a reparative effect on the cornea during epithelial injury (14). EGFR2/ErbB2 and EGFR3/ErbB3 have also been confirmed to be expressed in the corneal epithelium, sharing a distribution pattern similar to EGFR1. Among them, the EGFR2/ErbB2 receptor enhances the corneal epithelial wound healing process by activating the ERK and PI3K signaling pathways (15). While the role of EGFR3/ErbB3 has not been fully elucidated, the existence of specific antibody inhibitors for EGFR3/ErbB3 has been confirmed. Utilizing these inhibitors and genetic techniques, studies have demonstrated that EGFR3/ErbB3 signaling can assist in the migration of corneal epithelial cells (16, 17). It’s worth noting that EGFR4/ErbB4 is not expressed in the corneal epithelium (18).

Presently, seven EGFR ligands have been identified. In addition to EGF, six other endogenous ligands capable of binding to EGFR have been recognized, including heparin-binding EGF-like growth factor (HB-EGF), transforming growth factor-α (TGF-α), betacellulin (BTC), epiregulin, amphiregulin, and epigen. HB-EGF, integral in promoting growth and development, plays a crucial role, as evidenced by the fact that knockout mice perish shortly after birth (19). Functioning as a soluble transmembrane protein, HB-EGF binds to an additional domain of negatively charged polysaccharides, thereby enhancing *in vitro* cell adhesion and promoting corneal epithelial injury repair (20). A notable discovery in the study indicates that HB-EGF exhibits prolonged cell attachment compared to EGF, resulting in a sustained impact on wound healing following brief therapy (21). TGF-α, a member of the epidermal growth factor family, is produced by both epidermal cells and macrophages. It plays a crucial role in the repair of corneal epithelial injuries by initiating multiple signaling cascade reactions upon binding with the EGFR (18, 22). The bidirectional interaction facilitated by TGF-α between corneal epithelial cells and mesenchymal cells assumes a pivotal morphological role in both corneal development and tissue repair. Any disruption in this intricate interplay can result in ocular lesions. Notably, TGF-α knockout mice exhibit significant ocular abnormalities, characterized by corneal epithelial thinning, inflammation, and edema (23, 24). TGF-α also promotes the proliferation of corneal epithelial and stromal cells (25). Additionally, TGF-α stimulates EGFR, facilitating the internalization and recycling of ligand-receptor complexes (22). Conversely, overexpression of TGF-α has been observed to induce corneal damage by activating EGFR in both corneal epithelium and stroma. This pathological manifestation is evident through a reduction in the number of corneal epithelial cell layers, corneal epithelial degeneration, conjunctivalization of the cornea, inhibition of the expression of the corneal pigment protein Kera, and a marked decrease in fibrocollagen types I and V collagen. Simultaneously, TGF-α overexpression can lead to corneal opacity by upregulating α-SMA and Wnt5a, while downregulating Col1a1, Col1a2, and Col5a1 (25–28). Some *in vitro* analysis of BTC indicates that BTC can expedite corneal epithelial injury repair and may even possess advantages over EGF in promoting corneal epithelial injury repair (18). LSCs, primarily located at the corneal-scleral junction, possess lifelong self-renewal capabilities and can produce transient amplifying cells (TACs). During corneal epithelial injury repair, TACs migrate towards the corneal center, proliferate, and differentiate into corneal epithelial cells, thereby promoting the healing of corneal epithelial wounds (29). Research has shown that treating injured mouse eyes with BTC results in significant increases in the expression of putative stem cell markers, such as DNp63α, ABCB5 and CK14. This suggests that BTC accelerates corneal LSCs proliferation and enhances mouse corneal epithelial repair by phosphorylating erk1/2 (30, 31). Despite the efficacy of various EGFR ligands in *in vitro* settings, *in vivo* wound healing is uniquely facilitated by EGF among the seven mentioned ligands. EGF also stands out as the sole ligand in human tears with an EGFR concentration closely aligned with the ligand Kd (18). Furthermore, EGFR can be reactivated through various effectors, such as phospholipase D (PLD) and extracellular ATP, to foster the migration and proliferation of cells during the wound healing process (32). Transient receptor potential (TRP) non-selective cation channels constitute a superfamily, which contains 28 different genes, and widely distributed in corneal epithelial cells and endothelial cells, its expression in the corneal epithelial layer contributes to the maintenance of corneal transparency and barrier function of the corneal epithelium. Research has shown that TRPV1 stimulation also induces increases in the proliferation and migration of corneal epithelial cells and the release of IL-6 and IL-8, and reduces the formation and of corneal neovascularization (CNV) and scar through transactivation of the EGFR. Meanwhile, TRPC4 stimulates corneal epithelial proliferation and migration by transactivation of the EGFR. TRPV in corneal epithelium can also promote homeostasis under thermal stimulation (33, 34).

**Figure 2:**
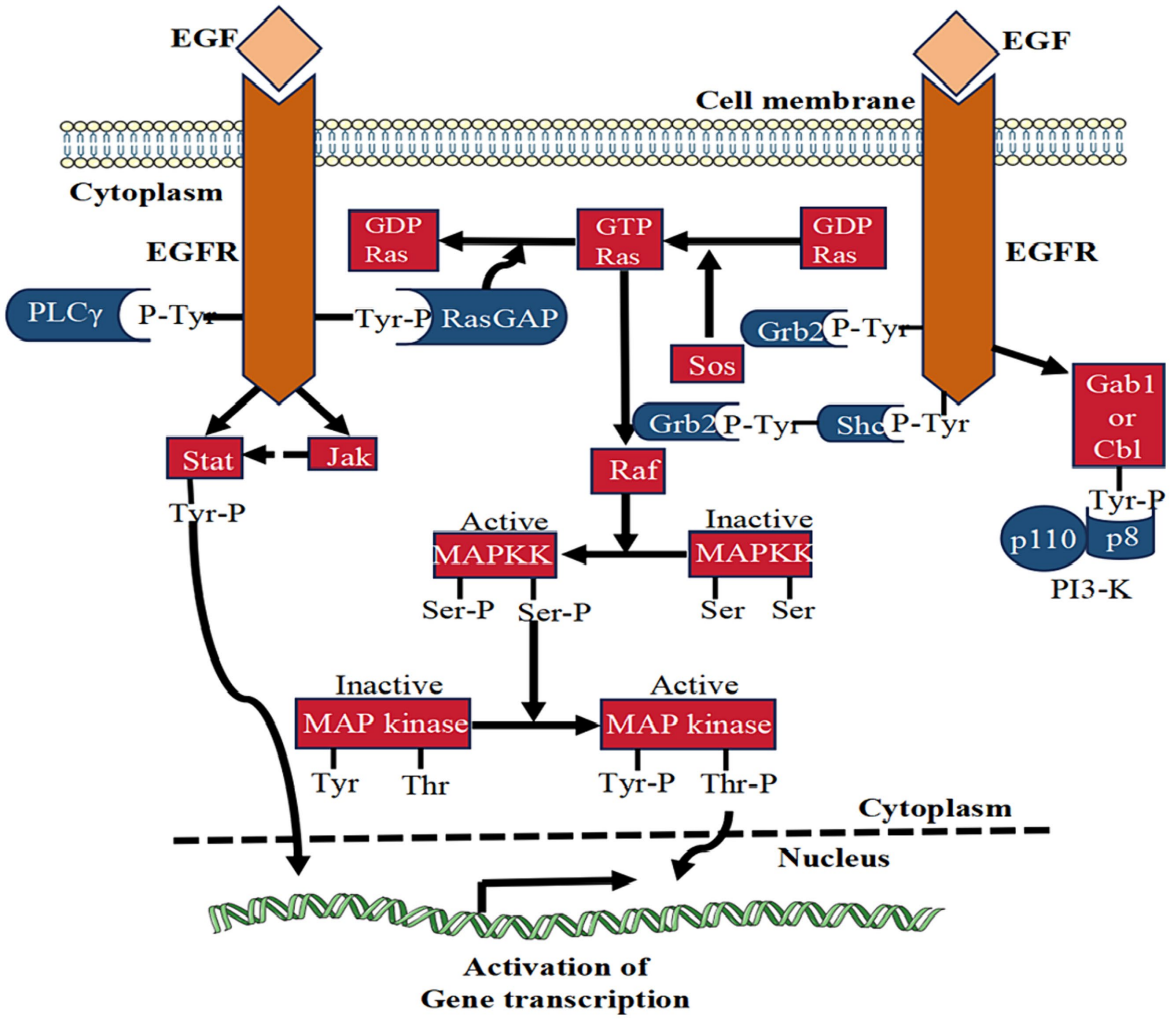
The signaling pathway of EGF. This figure illustrates the signal transduction mechanism of EGF in corneal epithelial injury repair. EGF binding activates EGFR, stimulating various signaling pathways like PLCγ, Ras-GAP, Grb2, and Shc. These pathways collectively contribute to the reparative effects on corneal epithelial injuries.

While EGF holds the potential to stimulate the migration and proliferation of corneal epithelial cells, caution is warranted, as excessively increasing the intensity and duration of EGF may not yield positive effects. An experiment assessing EGF’s effectiveness has revealed potential harm from continuous daily injections in rats (35). Furthermore, injecting recombinant EGF into the cornea post-corneal epithelial cell injury can lead to CNV (36). Elevated tear EGF levels are also associated with meibomian duct hypertrophy, contributing to meibomian gland hyperplasia (37). These findings underscore the need for caution when employing exogenous EGF in treating corneal injuries. Additionally, EGFR activity is a critical determinant in maintaining corneal epithelial homeostasis and plays a pivotal role in restoring damaged corneal epithelial cells. Despite the inherent regulatory mechanisms preventing sustained EGFR signal transduction, a deeper understanding of the molecular mechanisms governing EGFR signaling holds promise for developing new methods to overcome these regulatory barriers and enhance the efficacy of EGF (Figure 2 shows the EGF signaling pathway).

### 2.2 Hepatocyte growth factor

Hepatocyte growth factor (HGF) is a growth factor originating from fibroblasts, predominantly produced by mesenchymal cells, and expressed in various cell types, including corneal epithelial cells, keratocytes and endothelial cells. Its mode of action is paracrine, exerting its effects on adjacent cells (38). Structurally, HGF consists of α- and β-chains and serves as a mitogen and motility factor. In the cornea, HGF plays a significant role by binding to its receptor c-met and primarily participating in the proliferation, mitosis, and morphogenesis of corneal epithelial cells (39–41).

When the corneal epithelium undergoes damage, the expression of HGF in corneal epithelial cells and keratocytes is upregulated. This upregulation activates the signal mediators phosphatidylinositol of PI3K/Akt, phosphoprotein 70 ribosomal protein S6 kinase (p70s6K), and ERK. Consequently, it controls the cell cycle, promoting cell division and proliferation of corneal epithelial cells by triggering the activity of NF-κB. Simultaneously, it reverses the anti-proliferative effect of pro-inflammatory cytokines interleukin-1β (IL-1β) and TNF-α on these cells in the inflammatory environment, mediating corneal epithelial injury repair (42–46). HGF also exhibits wound repair effects by inhibiting the inflammatory response of corneal epithelial cells. Studies have demonstrated that HGF can inhibit the activation of immune cells and the expression of inflammatory factors. It further suppresses the expression of TNF-α, monocyte chemotactic protein-1 (MCP-1), and IL-6 in the macrophage system *in vitro*. Additionally, it promotes the production of the anti-inflammatory cytokine IL-10 in bone marrow-derived macrophages and dendritic cells stimulated by lipopolysaccharide (LPS) (43, 47–49). Evidence supports that HGF significantly inhibits cell apoptosis, temporarily downregulates the expression of cell cycle inhibitors in corneal epithelial cells, and upregulates cyclin and cyclin-dependent kinases. It also influences tumor suppressor proteins Rb and p53, which regulate cell cycle and apoptosis. Through these mechanisms, HGF actively participates in corneal epithelial injury repair (50, 51). HGF also has the capability to penetrate through LPS-induced corneal opacity, promoting recovery, diminishing corneal fibrosis, restoring normal corneal tissue structure, and reestablishing immune quiescence after keratitis (52). In cases of diabetes-related corneal epithelial damage, HGF exhibits a reparative effect by restoring the level of c-met in the cornea of diabetic patients through downstream activation of p38 mitogen-activated protein kinase (MAPK) and the production of several putative stem cell markers. Importantly, this positive effect is observed in cultured corneas, regardless of whether gene therapy is applied to the entire corneal epithelial cells or only to the corneal edge area containing stem cells (53). Research indicates that silencing the HGF gene inhibits corneal epithelial proliferation and UVR-induced CNV. Additionally, HGF contributes to the upregulation of vascular endothelial growth factor (VEGF) and plays a role in angiogenesis regulation. These findings open up new avenues for exploring treatment strategies for CNV (54, 55).

### 2.3 Insulin-like growth factor

Insulin-like growth factor (IGF) belongs to the multifunctional cell proliferation regulatory factor, representing a group of peptide substances capable of promoting growth. Its secretory cells are widely distributed in various tissues, including the liver, kidneys, heart and eyes of the human body. The IGF family comprises two peptide ligands (IGF-1 and IGF-2), three receptors, and six binding proteins, collectively maintaining tissue homeostasis by regulating metabolism and/or mitotic pathways at the level of all corneal cells (56).

IGF-1, a multifunctional cytokine with broad biological activity, holds considerable promise for applications in corneal epithelial injury repair. By binding to the insulin-like growth factor 1 receptor (IGF-1R), IGF-1 actively maintains and regulates corneal epithelial cell growth, proliferation, differentiation, maturation, migration, regeneration, and energy metabolism. It promotes corneal epithelial cell proliferation through the activation of the hybrid of IGF-1R and insulin receptor (INSR), leading to subsequent Akt phosphorylation. Additionally, IGF-1 mediates corneal epithelial cell migration through the PI3K/AKT pathway. Furthermore, IGF-1 promotes the expression of IGF receptors in corneal limbal cells, stimulating LSCs to differentiate into corneal epithelial cells (57, 58). Beyond its role in cell proliferation and migration, IGF-1 serves as a crucial neurotrophic factor facilitating the regeneration and restoration of nerves following peripheral nerve damage in the cornea (59). In combination with substance P, IGF-1 demonstrates a synergistic effect in promoting corneal epithelial injury repair. The co-application accelerates the *ex vivo* migration of corneal epithelial cells in the injured corneal stroma. Mediated by the interaction between substance P and tachykinin receptors, it enhances the adhesion of corneal epithelium to fibronectin (FN) and type IV collagen, thereby augmenting the protective role of the corneal epithelium through the stimulation of wound healing (60–64). The low levels of IGF-1 in tears, particularly the reduced proportion of IGF-1 and IGF binding protein 3 (IGFBP-3) in tears of diabetic patients, have been associated with decreased proliferation of corneal epithelial cells and delayed wound repair. This change inhibits the capacity of IGF-1 to induce IGF-1R or hybrid R phosphorylation (65, 66). Research has demonstrated that mRNA of adipose-derived stem cells (ADSCs) modified with IGF-1 exhibits stronger cell proliferation and migration abilities, promoting wound repair, morphological and functional recovery, corneal nerve regeneration, and maintenance of corneal homeostasis after acute alkali burns. Importantly, it can prevent the generation of CNV and corneal lymphatic vessels, highlighting the crucial role of IGF-1 in the repair of corneal epithelial injury (60). However, the application of IGF-1 protein to the cornea in the form of eye drops faces limitations, including a limited duration of effect, elevated attrition rates, and the need for repeated administration. Further research is expected to explore new carrier forms that can overcome these shortcomings and enhance the effectiveness of IGF-1 in corneal injury repair. The expression of IGF-2 and its receptors significantly increases after corneal epithelial cell injury, promoting the transformation of LSCs in the basal layer of the cornea into corneal epithelial cells and subsequently supporting corneal epithelial cell repair (67). Additionally, both IGF-1 and IGF-2 play roles in promoting the proliferation of keratocytes and collagen synthesis (68).

IGFBP primarily exists in the aqueous humor and vitreous body, exerting unique, cell- and tissue-dependent effects through interactions with the IGF family via binding (69–71). The primary function of IGFBP is to bind to IGF-1, extending its half-life in circulation and preventing IGF-1R activation induced by IGF-1 (72, 73). IGFBP-2 and IGFBP-3 play pivotal roles in corneal tissue homeostasis, particularly in regulating the growth of corneal epithelial cells and the localization of intracellular receptors (74, 75). The mutual regulation between IGFBP-3 and IGF-1R maintains corneal epithelial homeostasis. Previous studies have shown that IGFBP-3 is essential for inducing the transport of IGF-1R, and the absence of IGF-1R will downregulate IGFBP-3 in turn (76). During conditions such as hypoxia and hyperglycemia, the secretion of IGFBP-3 increases. For instance, the level of IGFBP-3 in the tears of diabetic patients rises, suggesting its potential role in regulating eye homeostasis in diabetic patients and indicating therapeutic potential in ocular surface diseases associated with diabetes (66).

Moreover, IGF and insulin share a close relationship, with the former mediating the action of insulin to promote the growth of corneal epithelial cells. This suggests a potential collaborative repair effect between the two, offering a promising avenue for future research exploration (77, 78).

### 2.4 Neurogenic growth factor

Neurogenic growth factor (NGF) belongs to the family of neurotrophic factors, exhibiting dual biological functions of neuronal nourishment and promoting synaptic growth (79). In the context of the cornea, signals mediated by NGF propagate through the high-affinity receptor tropomyosin receptor kinase A (TrkA) and the low-affinity non-selective transmembrane glycoprotein receptor p75NTR. When combined with NGF, TrkA activates Ras MAPK, ERK, phospholipase C-γ (PLC-γ), and PI3K. This activation includes stimulating D-type cell cycle regulatory proteins through PI3K/Akt and MAPK/ERK, subsequently promoting corneal epithelial cell cycle progression. Simultaneously, p75NTR activates the c-Jun kinase and NF-kB signaling pathway, exhibiting a protective effect on corneal epithelial cells by inhibiting the inflammatory signaling pathway of NF-kB (80–82).

Research has demonstrated that NGF participates in the repair process of corneal epithelial and stromal damage by upregulating matrix metalloproteinase-9 (MMP-9) and cleaving integrins β4 to stimulate the migration of corneal epithelial cells, promotes the differentiation of keratocytes into myofibroblasts, and reduces the formation of corneal haze (83–85). Moreover, NGF induces the differentiation of goblet cells and the production of mucin through receptors expressed in the lacrimal gland and neural reflexes, thereby contributing to the maintenance of corneal epithelial function (86). In addition to its role in cellular functions, NGF regulates immune function through Toll-like receptors (TLRs) in corneal physiology and pathology, playing a crucial role in maintaining corneal homeostasis both *in vivo* and *in vitro* settings (87). NGF has also been identified as a key promoter for the proliferation of LSCs, the formation of colonies in LSCs, and the maintenance of the LSC phenotype (79). For patients with corneal ulcers, local application of NGF eye drops has been shown to improve the speed of corneal epithelial repair and the sensitivity of the cornea. In cases of herpes simplex keratitis (HSK), endogenous NGF, akin to acyclovir, significantly improves the condition and inhibits recurrence. Clinical studies indicate that eye drops containing NGF can induce complete healing in HSK patients resistant to acyclovir (88, 89). Treatment with recombinant human NGF (rhNGF) has proven effective in enhancing corneal perception in patients with neurotrophic keratitis (NK) by increasing the density and number of nerve fibers in the basal layer of the corneal epithelium. It also promotes the healing of persistent corneal epithelial defects and ulcers. Furthermore, rhNGF provides lubrication and natural protection against pathogen damage to the corneal epithelium by promoting tear secretion from the lacrimal gland. RhNGF has received approval as a primary therapeutic drug for NK (87, 88). Additionally, NGF exhibits the ability to inhibit oxidative damage caused by hyperosmotic stress or high glucose levels. This finding suggests its potential therapeutic effect on conditions such as dry eye syndrome and diabetic keratopathy (DK) (90, 91).

### 2.5 TGF-β

TGF is a protein composed of amino acids in the cytoplasm, belonging to the family of peptide growth factors. It includes two main types: TGF-α and TGF-β (92). TGF-α has been described in the EGF section. TGF-β is a multifunctional growth factor, further divided into three subtypes: TGF-β1, TGF-β2 and TGF-β3. All three subtypes and their receptors are expressed in corneal epithelium and keratocytes (93). TGF-β assumes a pivotal role in orchestrating and coordinating the response to corneal injury repair, exerting influence over various facets such as the proliferation, motility, and differentiation of corneal epithelial cells. Moreover, TGF-β modulates the activity and apoptosis of keratocytes, as well as the development of myofibroblasts (94). By stimulating the migration of corneal epithelial cells through integrin β1, TGF-β enhances the fluidity of these cells (95). Notably, the conditional ablation of its type II receptor has been found to impede the repair of corneal epithelial wounds and the activation of p38 MAPK, thereby hindering the migration of corneal epithelial cells (96). Furthermore, TGF-β2 has been substantiated to expedite the repair of corneal epithelial wounds in rabbits, augmenting barrier integrity by promoting cell adhesion to substrates and enhancing the functionality of corneal endothelial cells (CECs) (97). Tgfbr-2 also plays a crucial role in maintaining corneal stromal homeostasis, as studies have demonstrated that Tgfbr-2 knockout mice display significant corneal thinning and a potential for corneal ectasia (98). TGF-β3 exhibits the capability to mitigate interstitial scars induced by the activity of TGF-β1 and TGF-β2. Moreover, it demonstrates potential therapeutic effects in addressing corneal and skin wounds in diabetic patients, acting through the PI3K-Akt and SMAD signaling pathways, along with their target genes (99). Additionally, if the Bowman layer is damaged, corneal cells are highly susceptible to exposure to TGF-β. In such cases, TGF-β promotes damage repair through various mechanisms (100). Despite its essential role in corneal epithelial injury repair, TGF-β also has negative effects on the cornea. For instance, it can promote the aging of corneal epithelial cells through the NF-κB signaling pathway. This aging process can be alleviated by inhibiting the NF-κB signaling pathway (101). TGF-β is implicated in the pathogenesis of various eye diseases, including pterygium, vernal keratoconjunctivitis (VKC), atopic keratoconjunctivitis (AKC), and graft-versus-host disease (GVHD). Elevated levels of TGF-β are observed in the corneas of individuals with these diseases (102). Additionally, TGF-β regulates the transformation of corneal epithelial cells and corneal fibroblasts into myofibroblasts, and the high expression of α-SMA and F-actin in myofibroblasts can lead to the loss of corneal transparency and corresponding corneal haze (103). Moreover, TGF-β1 and TGF-β2 can prevent corneal epithelial cells from proliferating *in vitro* (104).

### 2.6 Platelet-derived growth factor

Platelet-derived growth factor (PDGF), secreted by epithelial cells, endothelial cells and inflammatory cells, serves as a potent mitogenic factor, existing in diverse isoforms, namely PDGF-AA, PDGF-BB, PDGF-CC, PDGF-DD, and PDGF-AB (105). Featuring both α and β types of receptors, PDGF exerts its cellular effects by inducing the complex formation of α-tyrosine kinase receptors and β-tyrosine kinase receptors. This induction, in turn, triggers processes such as cell growth, chemotaxis, actin recombination and protection against apoptosis. Analogous to TGF-β, PDGF assumes a pivotal role in regulating and coordinating the response to corneal wound repair. It influences the proliferation, motility and differentiation of corneal epithelial cells, while also modulating the activity and apoptosis of keratocytes and contributing to the development of myofibroblasts (94). Corneal epithelial cells express PDGF AA, PDGF BB and PDGF AB, which regulate the migration and proliferation of keratocytes. In the presence of FN, these isoforms can enhance the migration of corneal epithelial cells (106–108). Research indicates that PDGF-AB and PDGF-BB promote the migration of corneal fibroblasts *in vitro*, leading to an increase in the concentration of cytosolic free Ca^2+^. PDGF-BB also significantly stimulates DNA synthesis in bovine corneal endothelial cells (BCEC) and human corneal fibroblasts (HCF) in a dose-dependent manner (108–110). Moreover, under high fibroblast density, PDGF isomers act as mitogens for interstitial fibroblasts during wound healing, conversely, at low cell density, PDGF-AA and PDGF-AB can prevent cell loss during the corneal homeostasis process (111). The secretion of PDGF is activated during corneal trauma, infection, or inflammation, providing significant stimulation for tissue repair. However, hyperstimulation can have negative effects. For instance, PDGF-α hyperstimulation can promote the proliferation and migration of lens epithelial cells, leading to epithelial-mesenchymal transition (EMT) (112, 113).

### 2.7 Fibroblast growth factor

Fibroblast growth factor (FGF) is secreted by the hypothalamus and pituitary gland, serving as a broad-spectrum mitogen, currently, at least 23 FGF families have been identified, stimulating or maintaining specific cellular functions required for tissue metabolism, homeostasis and development through signaling axes mediated by their receptors (114). Corneal epithelial changes, accompanied by decreased vision and dry eye symptoms, have been observed after treatment with inhibitors of the FGF receptor (FGFR), indirectly indicating FGF’s involvement in corneal epithelial homeostasis (115). Basic FGF (b-FGF/FGF2), approved for the treatment of corneal damage, accelerates the repair of corneal epithelial cell damage and reduces keratitis by promoting the proliferation, differentiation and migration of corneal epithelial cells (116, 117). In experiments involving FGFR2 knockout mice, observations reveal localized central corneal thinning, along with the loss of collagen fibers and apoptosis of keratocytes (118). In addition, the signal transduction of FGFR2b promotes corneal epithelial injury repair, studies have found that FGFR2b knockout mice exhibit reduced proliferation of corneal epithelial cells, as well as loss of lacrimal gland and meibomian gland. FGFR2b is also necessary for the development of submandibular glands (119). FGF-10 plays a crucial role in the development of the cornea, morphogenesis and growth of the lens and induction and branching of the lacrimal gland and meibomian gland. Research has shown that FGF-10 is essential for the development of lacrimal glands in humans and mice (120, 121). FGF-10 can upregulate the expression of mucin in conjunctival epithelial cells, protecting the ocular surface in a dry eye model of rabbits and controlling the migration of epithelial cells during the process of embryonic eyelid closure (122, 123). Additionally, FGF-10 is associated with adult tissue homeostasis and the function of stem cells (124). In an experimental study of DK, it has been found that rhFGF-21 can improve the vitality and migration of human corneal epithelial cells, promote the healing of corneal wounds and the production of tears, and improve corneal edema. RhFGF-21 significantly reduces the expression of pro-inflammatory cytokines such as TNF-α and MMPs in corneal epithelial cells, while increasing the level of anti-inflammatory molecules IL-10 and SOD-1. RhFGF-21 also inhibits excessive production of reactive oxygen species (ROS) and alleviates oxidative stress induced by hyperglycemia in corneal epithelial cells. Therefore, the application of drugs containing FGF-21 may be a potential treatment method for DK (125, 126).

### 2.8 Keratinocyte growth factor

Keratinocyte growth factor (KGF) belongs to the FGF family, officially known as FGF-7. It is produced by mesenchymal cells and acts on adjacent cells in a paracrine manner. KGF promotes the repair of corneal epithelial wounds through the signaling cascade of MAPK and PI3K/p70 S6 in corneal epithelial cells. It also inhibits the destruction of the barrier function caused by hypoxia in corneal epithelial cells by activating ERK (46, 127, 128). *In vitro* experiments have shown that KGF can protect cells from apoptosis for an extended period, with the final percentage of apoptosis in cells treated with KGF being only 10% (129). Similar to HGF, KGF has the capacity to inhibit UVR-induced corneal epithelial proliferation. Through gene silencing, it downregulates the expression of VEGF and its receptors, consequently mitigating CNV (48, 49). Additionally, KGF-2 exhibits certain effects in re-epithelialization, accelerating migration, reducing scar formation and edema. It is considered superior to b-FGF and holds potential as a new drug for treating corneal injuries (116). KGF also can promote the migration of LSCs, thereby promoting the repair of corneal epithelial damage (36).

### 2.9 Opioid growth factor

Opioid growth factor (OGF) is an endogenous peptide found together with its receptor OGFr in or on the basal layer of many species’ corneas, when combined, OGF regulates DNA synthesis in corneal epithelial cells and influences cell migration (130). Experimental evidence suggests that OGF inhibits cell overgrowth by upregulating cyclin-dependent inhibitory kinases p16 and p21, contributing to the maintenance of corneal epithelial homeostasis (131). Studies conducted on patients and rats with diabetes have indicated elevated levels of OGF and OGFr in serum and corneal epithelium. This elevation has been associated with ocular surface complications, including dry eyes, abnormal sensitivity of the corneal surface, and delayed corneal epithelial repair. In rats, an OGF-OGFr axis is present in the corneal limbus, and its dysregulation in hyperglycemia impacts the morphology of the corneal limbus, exacerbating diabetes-related complications on the corneal surface. Local application of the opioid antagonist naltrexone (NTX) has demonstrated improvement in this situation (132, 133). NTX disrupts the OGF-OGFr interaction, resulting in increased DNA synthesis in epithelial cells of the peripheral cornea and limbus corneae, as well as the proliferation of fibroblast cells. The latter plays a crucial role in corneal wound healing (134, 135). Additionally, the application of NTX significantly promotes corneal re-epithelialization and increases tear production (136–138). In summary, the use of eye drops containing opioid antagonists, such as NTX, holds promise as a novel therapy for treating wound repair disorders of the corneal epithelium.

### 2.10 VEGF

**Figure 3:**
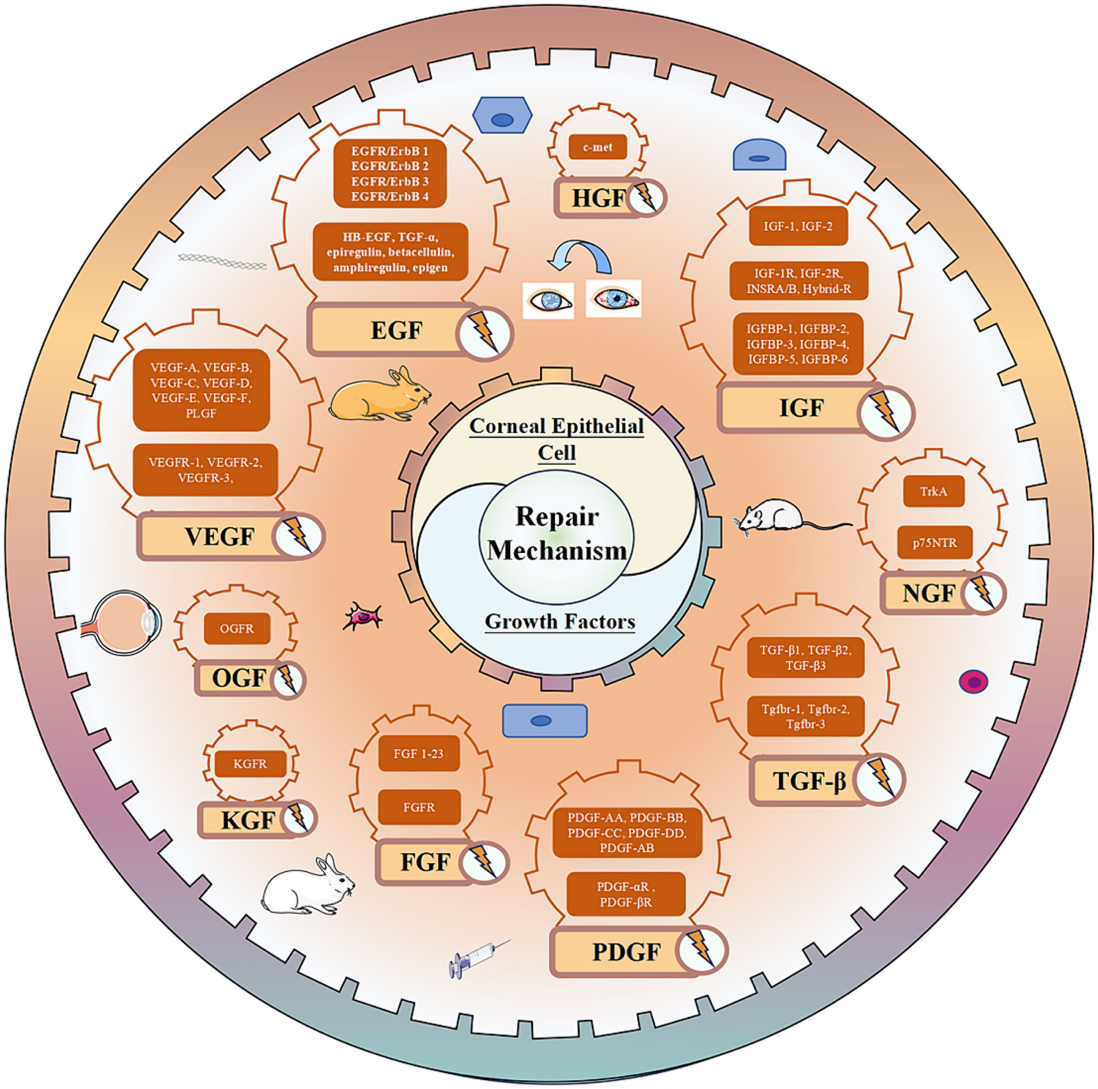
Growth factor mediated repair mechanism of corneal epithelial cell injury.

VEGF, recognized as a highly specific mitogen promoting endothelial cell growth, is also referred to as vascular permeability factor (VPF) due to its ability to significantly enhance vascular permeability (139). The VEGF family comprises seven subtypes: VEGF-A, VEGF-B, VEGF-C, VEGF-D, VEGF-E, VEGF-F, and placental growth factor (PLGF). Additionally, there are three receptors, VEGFR-1, VEGFR-2, and VEGFR-3. Among these, VEGF-A stands out for its profound ability to promote angiogenesis and is the most prevalent subtype in the eyes (140). The VEGF family and its receptors are expressed in the corneal epithelium (4). Under normal circumstances, a delicate balance is maintained between ocular angiogenic factors and anti-angiogenic factors to prevent pathological CNV production. However, factors such as wearing contact lenses, inflammation, and infection can disrupt this balance and lead to CNV (141). Research has highlighted the crucial role of VEGF in the pathogenesis of CNV, making the use of anti-VEGF drugs a feasible therapeutic approach (142, 143). Additionally, VEGF is implicated in non-angiogenic functions, such as neuroprotection and serving as a nutritional factor for corneal nerves (144). VEGF also plays a role in wound repair; studies have shown that VEGF accelerates corneal epithelial wound healing by stimulating corneal nerve regeneration (145). VEGF may be linked to the pathogenesis of pterygium, with higher expression detected in pterygium compared to normal tissue (146). Pigment epithelial-derived factors (PEDFs) are closely related to VEGF, sharing anti-angiogenic functions and a protective role for corneal nerves (147). Both factors exhibit synergistic therapeutic effects in certain diseases. However, in pterygium, a decrease in PEDF expression has been observed (146). Moreover, PEDF has been found to promote the self-renewal and migration of LSCs, thereby facilitating corneal epithelial repair (148). (The expression of growth factors in the corneal epithelium is depicted in Figure 3, and the biological effects of corneal epithelial growth factor receptors are summarized in Table 1).

**Table 1:** Biological effects of corneal epithelial growth factor receptor.

Factor	Receptor	Effects on corneal wound healing	References
EGF	EGFR	Promotes migration and proliferation of epithelial cells EGFR can be reactivated through various effectors	(7, 8, 12, 13, 17, 32)
HB-EGF		Enhances cell adhesion	(19)
BTC		Accelerates corneal epithelial injury repair Accelerates the proliferation of LSCs	(18, 30)
TGF-α		Promotes the proliferation of corneal epithelial and stromal cells Facilitates the internalization and recycling of ligand-receptor complexes	(22, 25)
HGF	c-Met	Participates in the mitosis and morphogenesis of corneal epithelial cells Promotes cell division and proliferation of corneal epithelial cells by controlling the cell cycle Reverses the anti-proliferative effect of pro-inflammatory cytokines in the inflammatory environment Inhibits the activation of immune cells and the expression of inflammatory factors Promotes the production of anti-inflammatory cytokines Suppresses apoptosis of corneal epithelial cells Promotes corneal opacity recovery, reduces corneal fibrosis, normalize corneal tissue structure and reestablish immune quiescence post-keratitis Restores the level of c-met in the cornea of diabetic patients Inhibits UVR-induced corneal epithelial proliferation and CNV by HGF gene silencing Participates in angiogenesis	(39–55)
IGF/INS	IGF-1R、IGF-2R、INSR、Hybrid-R	Regulates metabolism and/or mitotic pathways Promotes the migration and proliferation of epithelial cells Facilitates the regeneration and restoration of corneal nerves Accelerates the *ex vivo* migration of corneal epithelial cells in the injured corneal stroma Enhances the adhesion of corneal epithelium to FN and type IV collagen Promotes the transformation of LSCs in the basal layer of the cornea into corneal epithelial cells Promotes the proliferation of keratocytes and collagen synthesis Plays pivotal roles in corneal tissue homeostasis	(56–64, 67, 68, 74, 75)
NGF	TrkA, p75NTR	Stimulates the migration of corneal epithelial cells, promotes the differentiation of keratocytes into myofibroblasts and reduces the formation of corneal haze Induces the differentiation of goblet cells and the production of mucin regulates immune function Be identified as a key promoter for the proliferation of LSCs, the formation of colonies in LSCs and the maintenance of the LSCs phenotype Improves the speed of corneal epithelial repair and the sensitivity of the cornea of patients with corneal ulcers, significantly improves the condition and inhibits recurrence of HSK Increases the density and number of nerve fibers in the basal layer of the corneal epithelium, promotes tear secretion Inhibits oxidative damage caused by hyperosmotic stress or high glucose levels	(79, 83–91, 149, 150)
TGF-β	Tgfbr-1, Tgfbr-2, Tgfbr-3	Modulates the activity and apoptosis of keratocytes, modulates the development of myofibroblasts Stimulates the migration of corneal epithelial cells Augments barrier integrity by promoting cell adhesion to substrates and enhancing the functionality of corneal endothelial cells (CECs) Plays an important role in corneal stromal homeostasis Reduces interstitial scars promotes damage repair of Bowman layer Promotes the aging of corneal epithelial cells Be implicated in the pathogenesis of various eye diseases, such as pterygium Regulates the transformation of corneal epithelial cells and corneal fibroblasts into myofibroblasts Prevents corneal epithelial cells from proliferating *in vitro*	(94–103)
(PDGF)	PDGF-αR, PDGF-βR	Promotes the proliferation, motility and differentiation of corneal epithelial cells Modulates the activity and apoptosis of keratocytes and contributes to the development of myofibroblasts Stimulates DNA synthesis in BCEC and HCF Acts as mitogens for interstitial fibroblasts during wound healing Prevents cell loss during the corneal homeostasis process	(94, 105–110)
FGF	FGFR	Participates in corneal epithelial homeostasis Promotes the proliferation, differentiation and migration of corneal epithelial cells Promotes the regeneration of the lacrimal gland, submandibular gland and meibomian gland Promotes the development of the cornea and morphogenesis and growth of the lens Be associated with adult tissue homeostasis and the function of stem cells Improves the vitality of human corneal epithelial cells Improves corneal edema Reduces the expression of pro-inflammatory cytokines and increases the level of anti-inflammatory molecules Suppresses excessive production of ROS and alleviates oxidative stress	(114–120, 123–125)
(KGF)	KGFR	Inhibits the destruction of the barrier function caused by hypoxia in corneal epithelial cells Suppresses apoptosis of corneal epithelial cells Inhibits UVR-induced corneal epithelial proliferation and CNV by HGF gene silencing Enhances re-epithelialization rates Promotes the migration and proliferation of epithelial cells Reduces corneal scars and edema Promotes the proliferation of LSCs	(36, 48, 49, 115, 127, 128)
OGF	OGFr	Regulates DNA synthesis in corneal epithelial cells and influences the migration of corneal cells Inhibits the overgrowth of corneal cells and maintains of corneal epithelial homeostasis	(129, 130)
VEGF	VEGFR-1, VEGFR-2, VEGFR-3	Promotes the growth of endothelial cell Promote corneal angiogenesis Protecting corneal nerves Be related to the pathogenesis of pterygium	(138, 139, 143, 145)

EGF, epidermal growth factor; EGFR, epidermal growth factor receptor; HB-EGF, heparin-binding EGF-like growth factor; BTC, betacellulin; TGF-α, transforming growth factor-α; HGF, hepatocyte growth factor; c-Met, hepatocyte growth factor receptor; IGF, insulin-like growth factor; INS, insulin; IGF-1R, insulin-like growth factor-1 receptor; IGF-2R, insulin-like growth factor-2 receptor; INSR, insulin receptor; Hybrid-R, IGF-1R and INSR hybrid; NGF, nerve growth factor; TrkA, tropomyosin receptor kinase A; p75NTR, low-affinity non-selective transmembrane glycoprotein receptor; TGF-β, transforming growth factor-β; Tgfbr-1, transforming growth factor beta receptor-1; Tgfbr-2, transforming growth factor beta receptor-2; Tgfbr-3, transforming growth factor beta receptor-3; PDGF, platelet derived growth factor; PDGF-αR, platelet derived growth factor receptor-α; PDGF-βR, platelet derived growth factor receptor-β; FGF, fibroblast growth factor; FGFR, fibroblast growth factor receptor; KGF, keratinocyte growth factor; KGFR, keratinocyte growth factor receptor; OGF, opioid growth factor; OGFr, opioid growth factor receptor.

## 3 Discussion and outlook

Eye injuries often involve damage to the corneal epithelial layer, leading to symptoms such as eye pain, bleeding, ulcers, and vision loss, significantly impacting quality of life (151, 152). The repair of corneal epithelial injuries has emerged as a prominent research focus, with growing recognition of the crucial role played by growth factors in this process. These growth factors contribute to the wound healing of corneal epithelium through intricate mechanisms.

Despite the significant role of growth factors in corneal epithelial injury repair, there are existing limitations. The current understanding of the signal transduction pathways of various growth factors is not yet comprehensive, and their full potential as a treatment method for corneal epithelial injury remains to be realized. Some growth factors, like TGF, have stringent usage and dosage guidelines in corneal epithelium treatment—only within a safe usage range can they effectively repair the corneal epithelium. This presents a crucial and challenging aspect in utilizing growth factors for corneal epithelium treatment. For targeted repair effects of growth factors, many studies focus on single targets or signal pathways. The comprehensive repair mechanisms involving different growth factors still require further exploration. Additionally, some growth factors are primarily limited to basic research, and their potential for improving corneal epithelial cells in clinical practice needs further validation. Growth factors can be categorized into endogenous and exogenous types, with exogenous growth factors often utilized in experimental studies involving mice. However, when it comes to the role of growth factors in corneal epithelial injury repair, there is a noticeable gap in research and discussion regarding whether the mechanisms differ between the two types. Furthermore, in the case of the growth factor VEGF, exploring more suitable drug carriers could potentially enhance its therapeutic efficacy. This avenue of research could lead to the development of more effective delivery systems for VEGF, optimizing its impact on corneal epithelial repair.

Future research endeavors should leverage current multi-omics techniques to explore and study the signaling mechanisms and synergistic effects of different growth factors, aiming to enhance their roles in corneal epithelial injury repair. Simultaneously, through in-depth basic and clinical research, optimizing the dosage and understanding side effects of growth factors can contribute to revealing their basic mechanisms and optimal usage methods. This foundational work will pave the way for the development of new treatment methods involving growth factors. Further research and exploration are essential to determine potential differences in the roles of endogenous and exogenous growth factors in corneal epithelial repair, a facet not addressed in the current study. Additionally, investigating ways to enhance exogenous growth factors and identifying more suitable drug carriers are critical for optimizing the use of growth factors in the future.

## Author contributions

JG: Writing – original draft. GD: Writing – review & editing. ZH: Writing – review & editing. YL: Writing – review & editing. AD: Writing – review & editing. CZ: Writing – review & editing.

## Glossary

EGF: Epidermal growth factor
EGFR/ErbB: Epidermal growth factor receptor
EGFR1/ErbB1: Epidermal growth factor receptor 1
PI3K: Type III phosphoinositide 3-kinase
Akt: Protein kinase B
ERK: Extracellular signal-regulated kinase
NF-kB: Nuclear factor kappa-B
HDAC6: Histone deacetylase 6
CTCF: CCCTC binding factor
PAX6: Paired box gene 6
EGFR2/ErbB2: Epidermal growth factor receptor 2
EGFR3/ErbB3: Epidermal growth factor receptor 3
EGFR4/ ErbB4: Epidermal growth factor receptor 4
HB-EGF: Heparin-binding EGF-like growth factor
TGF-α: Transforming growth factor-α
BTC: Betacellulin
PLD: Phospholipase D
CNV: Corneal neovascularization
HGF: Hepatocyte growth factor
p70s6K: Phosphoprotein 70 ribosomal protein S6 kinase
IL-1β: Interleukin-1β
MCP-1: Monocyte chemotactic protein-1
LPS: Lipopolysaccharide
MAPK: Mitogen-activated protein kinase
VEGF: Vascular endothelial growth factor
IGF: Insulin-like growth factor
IGFBP: IGF binding protein
IGF-1R: Insulin-like growth factor 1 receptor
INSR: Insulin receptor
LSCs: Corneal LSCs
FN: Fibronectin
IGFBP-3: IGF binding protein-3
ADSCs: Adipose-derived stem cells
IGFBP-2: IGF binding protein-2
TrkA: Tropomyosin receptor kinase A
PLC-γ: Phospholipase C-γ
MMP-9: Matrix metalloproteinase-9
TLRs: Toll-like receptors
HSK: Herpes simplex keratitis
rhNGF: Recombinant human NGF
NK: Neurotrophic keratitis
DK: Diabetic keratopathy
TGF: Transforming growth factor
CECs: Corneal endothelial cells
VKC: Vernal keratoconjunctivitis
AKC: Atopic keratoconjunctivitis
GVHD: Graft-versus-host disease
PDGF: Platelet-derived growth factor
BCEC: Bovine corneal endothelial cells
HCF: Human corneal fibroblasts
EMT: Epithelial-mesenchymal transition
FGF: Fibroblast growth factor
FGFR: Fibroblast growth factor receptor
b-FGF/FGF2: Basic FGF
ROS: Reactive oxygen species
KGF: Keratinocyte growth factor
OGF: Opioid growth factor
OGFr: Opioid growth factor receptor
NTX: Naltrexone

## References

[1] Ludwig PE Lopez MJ Sevensma KE. Anatomy, head and neck, eye cornea In: StatPearls. Treasure Island, FL: StatPearls Publishing (2023). 29262108

[2] Downie LE Bandlitz S Bergmanson JPG Craig JP Dutta D Maldonado-Codina C. CLEAR—anatomy and physiology of the anterior eye. Cont Lens Anterior Eye. (2021) 44:132–56. doi: 10.1016/j.clae.2021.02.009, PMID: 33775375

[3] Koh S Rao SK Srinivas SP Tong L Young AL. Evaluation of ocular surface and tear function—a review of current approaches for dry eye. Indian J Ophthalmol. (2022) 70:1883–91. doi: 10.4103/ijo.IJO_1804_21, PMID: 35647953 PMC9359282

[4] Tarvestad-Laise KE Ceresa BP. Modulating growth factor receptor signaling to promote corneal epithelial homeostasis. Cells. (2023) 12:2730. doi: 10.3390/cells12232730, PMID: 38067157 PMC10706396

[5] Klenkler B Sheardown H Jones L. Growth factors in the tear film: role in tissue maintenance, wound healing, and ocular pathology. Ocul Surf. (2007) 5:228–39. doi: 10.1016/s1542-0124(12)70613-4, PMID: 17660896

[6] Shin SH Koh YG Lee WG Seok J Park KY. The use of epidermal growth factor in dermatological practice. Int Wound J. (2023) 20:2414–23. doi: 10.1111/iwj.14075, PMID: 36584669 PMC10333026

[7] Zieske JD Takahashi H Hutcheon AE Dalbone AC. Activation of epidermal growth factor receptor during corneal epithelial migration. Invest Ophthalmol Vis Sci. (2000) 41:1346–55. PMID: 10798649

[8] Wilson SE. Corneal wound healing. Exp Eye Res. (2020) 197:108089. doi: 10.1016/j.exer.2020.108089, PMID: 32553485 PMC7483425

[9] Zhang Y Akhtar RA. Epidermal growth factor stimulation of phosphatidylinositol 3-kinase during wound closure in rabbit corneal epithelial cells. Invest Ophthalmol Vis Sci. (1997) 38:1139–48. PMID: 9152233

[10] Yang L Di G Qi X Qu M Wang Y Duan H. Substance P promotes diabetic corneal epithelial wound healing through molecular mechanisms mediated via the neurokinin-1 receptor. Diabetes. (2014) 63:4262–74. doi: 10.2337/db14-0163, PMID: 25008176

[11] Xu K Yu FS. Impaired epithelial wound healing and EGFR signaling pathways in the corneas of diabetic rats. Invest Ophthalmol Vis Sci. (2011) 52:3301–8. doi: 10.1167/iovs.10-5670, PMID: 21330660 PMC3109029

[12] Li T Lu L. Epidermal growth factor-induced proliferation requires down-regulation of Pax 6 in corneal epithelial cells. J Biol Chem. (2005) 280:12988–95. doi: 10.1074/jbc.M412458200, PMID: 15659382

[13] Imanishi J Kamiyama K Iguchi I Kita M Sotozono C Kinoshita S. Growth factors: importance in wound healing and maintenance of transparency of the cornea. Prog Retin Eye Res. (2000) 19:113–29. doi: 10.1016/s1350-9462(99)00007-5, PMID: 10614683

[14] Liu Z Carvajal M Carraway CA Carraway K Pflugfelder SC. Expression of the receptor tyrosine kinases, epidermal growth factor receptor, ErbB2, and ErbB3, in human ocular surface epithelia. Cornea. (2001) 20:81–5. doi: 10.1097/00003226-200101000-00016, PMID: 11189010

[15] Xu KP Riggs A Ding Y Yu FS. Role of ErbB2 in corneal epithelial wound healing. Invest Ophthalmol Vis Sci. (2004) 45:4277–83. doi: 10.1167/iovs.04-0119, PMID: 15557433 PMC2666385

[16] Huang J Wang S Lyu H Cai B Yang XH Wang J. The anti-ErbB3 antibody MM-121/SAR256212 in combination with trastuzumab exerts potent antitumor activity against trastuzumab-resistant breast cancer cells. Mol Cancer. (2013) 12:134. doi: 10.1186/1476-4598-12-134, PMID: 24215614 PMC3829386

[17] Schoeberl B Faber AC Li D Liang MC Crosby K Onsum M. An ErbB3 antibody, MM-121, is active in cancers with ligand-dependent activation. Cancer Res. (2010) 70:2485–94. doi: 10.1158/0008-5472.CAN-09-3145, PMID: 20215504 PMC2840205

[18] Peterson JL Phelps ED Doll MA Schaal S Ceresa BP. The role of endogenous epidermal growth factor receptor ligands in mediating corneal epithelial homeostasis. Invest Ophthalmol Vis Sci. (2014) 55:2870–80. doi: 10.1167/iovs.13-12943, PMID: 24722692 PMC4008048

[19] Iwamoto R Yamazaki S Asakura M Takashima S Hasuwa H Miyado K. Heparin-binding EGF-like growth factor and ErbB signaling is essential for heart function. Proc Natl Acad Sci USA. (2003) 100:3221–6. doi: 10.1073/pnas.0537588100, PMID: 12621152 PMC152273

[20] Block ER Matela AR Sundar Raj N Iszkula ER Klarlund JK. Wounding induces motility in sheets of corneal epithelial cells through loss of spatial constraints: role of heparin-binding epidermal growth factor-like growth factor signaling. J Biol Chem. (2004) 279:24307–12. doi: 10.1074/jbc.M401058200, PMID: 15039441

[21] Tolino MA Block ER Klarlund JK. Brief treatment with heparin-binding EGF-like growth factor, but not with EGF, is sufficient to accelerate epithelial wound healing. Biochim Biophys Acta. (2011) 1810:875–8. doi: 10.1016/j.bbagen.2011.05.011, PMID: 21640162 PMC3143286

[22] Roepstorff K Grandal MV Henriksen L Knudsen SLJ Lerdrup M Grøvdal L. Differential effects of EGFR ligands on endocytic sorting of the receptor. Traffic. (2009) 10:1115–27. doi: 10.1111/j.1600-0854.2009.00943.x, PMID: 19531065 PMC2723868

[23] Singh B Coffey RJ. From wavy hair to naked proteins: the role of transforming growth factor alpha in health and disease. Semin Cell Dev Biol. (2014) 28:12–21. doi: 10.1016/j.semcdb.2014.03.003, PMID: 24631356 PMC4105142

[24] Luetteke NC Qiu TH Peiffer RL Oliver P Smithies O Lee DC. TGF alpha deficiency results in hair follicle and eye abnormalities in targeted and waved-1 mice. Cell. (1993) 73:263–78. doi: 10.1016/0092-8674(93)90228-i, PMID: 8477445

[25] Zhang L Yuan Y Yeh LK Dong F Zhang J Okada Y. Excess transforming growth factor-α changed the cell properties of corneal epithelium and stroma. Invest Ophthalmol Vis Sci. (2020) 61:20. doi: 10.1167/iovs.61.8.20, PMID: 32668000 PMC7425719

[26] Reneker LW Silversides DW Xu L Overbeek PA. Formation of corneal endothelium is essential for anterior segment development—a transgenic mouse model of anterior segment dysgenesis. Development. (2000) 127:533–42. doi: 10.1242/dev.127.3.533, PMID: 10631174

[27] Liu CY Shiraishi A Kao CW Converse RL Funderburgh JL Corpuz LM. The cloning of mouse keratocan cDNA and genomic DNA and the characterization of its expression during eye development. J Biol Chem. (1998) 273:22584–8. doi: 10.1074/jbc.273.35.22584, PMID: 9712886

[28] Chen S Mienaltowski MJ Birk DE. Regulation of corneal stroma extracellular matrix assembly. Exp Eye Res. (2015) 133:69–80. doi: 10.1016/j.exer.2014.08.001, PMID: 25819456 PMC4379422

[29] Lehrer MS Sun TT Lavker RM. Strategies of epithelial repair: modulation of stem cell and transit amplifying cell proliferation. J Cell Sci. (1998) 111:2867–75. doi: 10.1242/jcs.111.19.2867, PMID: 9730979

[30] Jeong WY Yoo HY Kim CW. β-cellulin promotes the proliferation of corneal epithelial stem cells through the phosphorylation of erk1/2. Biochem Biophys Res Commun. (2018) 496:359–66. doi: 10.1016/j.bbrc.2018.01.054, PMID: 29331377

[31] Seyed-Safi AG Daniels JT. The limbus: structure and function. Exp Eye Res. (2020) 197:108074. doi: 10.1016/j.exer.2020.108074 32502532

[32] Zhang F Yang H Pan Z Wang Z Wolosin JM Gjorstrup P. Dependence of resolvin-induced increases in corneal epithelial cell migration on EGF receptor transactivation. Invest Ophthalmol Vis Sci. (2010) 51:5601–9. doi: 10.1167/iovs.09-4468, PMID: 20538990 PMC3061499

[33] Pan Z Yang H Reinach PS. Transient receptor potential (TRP) gene superfamily encoding cation channels. Hum Genomics. (2011) 5:108–16. doi: 10.1186/1479-7364-5-2-108, PMID: 21296744 PMC3525231

[34] Yang Y Yang H Wang Z Okada Y Saika S Reinach PS. Wakayama symposium: dependence of corneal epithelial homeostasis on transient receptor potential function. Ocul Surf. (2013) 11:8–11. doi: 10.1016/j.jtos.2012.09.001, PMID: 23321353

[35] Hennessey PJ Nirgiotis JG Shinn MN Andrassy RJ. Continuous EGF application impairs long-term collagen accumulation during wound healing in rats. J Pediatr Surg. (1991) 26:362–6. doi: 10.1016/0022-3468(91)90980-8, PMID: 1647451

[36] Nezu E Ohashi Y Kinoshita S Manabe R. Recombinant human epidermal growth factor and corneal neovascularization. Jpn J Ophthalmol. (1992) 36:401–6. PMID: 1289616

[37] Rao K Farley WJ Pflugfelder SC. Association between high tear epidermal growth factor levels and corneal subepithelial fibrosis in dry eye conditions. Invest Ophthalmol Vis Sci. (2010) 51:844–9. doi: 10.1167/iovs.09-3875, PMID: 19815739 PMC2868446

[38] Bottaro DP Rubin JS Faletto DL Chan AML Kmiecik TE Vande Woude GF. Identification of the hepatocyte growth factor receptor as the c-met proto-oncogene product. Science. (1991) 251:802–4. doi: 10.1126/science.1846706 1846706

[39] Pai P Kittur SK. Hepatocyte growth factor: a novel tumor marker for breast cancer. J Cancer Res Ther. (2023) 19:S0. doi: 10.4103/jcrt.JCRT_1084_16, PMID: 37147943

[40] Nakamura T Nishizawa T Hagiya M Seki T Shimonishi M Sugimura A. Molecular cloning and expression of human hepatocyte growth factor. Nature. (1989) 342:440–3. doi: 10.1038/342440a0 2531289

[41] Wright JW Church KJ Harding JW. Hepatocyte growth factor and macrophage-stimulating protein “hinge” analogs to treat pancreatic cancer. Curr Cancer Drug Targets. (2019) 19:782–95. doi: 10.2174/1568009619666190326130008, PMID: 30914029

[42] Chandrasekher G Kakazu AH Bazan HE. HGF- and KGF-induced activation of PI-3K/p 70 s6 kinase pathway in corneal epithelial cells: its relevance in wound healing. Exp Eye Res. (2001) 73:191–202. doi: 10.1006/exer.2001.1026, PMID: 11446769

[43] Omoto M Suri K Amouzegar A Li M Katikireddy KR Mittal SK. Hepatocyte growth factor suppresses inflammation and promotes epithelium repair in corneal injury. Mol Ther. (2017) 25:1881–8. doi: 10.1016/j.ymthe.2017.04.020, PMID: 28502469 PMC5542635

[44] Okunishi K Dohi M Fujio K Nakagome K Tabata Y Okasora T. Hepatocyte growth factor significantly suppresses collagen-induced arthritis in mice. J Immunol. (2007) 179:5504–13. doi: 10.4049/jimmunol.179.8.5504, PMID: 17911637

[45] Benkhoucha M Santiago-Raber ML Schneiter G Chofflon M Funakoshi H Nakamura T. Hepatocyte growth factor inhibits CNS autoimmunity by inducing tolerogenic dendritic cells and CD25^+^ Foxp3^+^ regulatory T cells. Proc Natl Acad Sci USA. (2010) 107:6424–9. doi: 10.1073/pnas.0912437107, PMID: 20332205 PMC2851995

[46] Giannopoulou M Dai C Tan X Wen X Michalopoulos GK Liu Y. Hepatocyte growth factor exerts its anti-inflammatory action by disrupting nuclear factor-kappa B signaling. Am J Pathol. (2008) 173:30–41. doi: 10.2353/ajpath.2008.070583, PMID: 18502824 PMC2438283

[47] Kusunoki H Taniyama Y Otsu R Rakugi H Morishita R. Anti-inflammatory effects of hepatocyte growth factor on the vicious cycle of macrophages and adipocytes. Hypertens Res. (2014) 37:500–6. doi: 10.1038/hr.2014.41, PMID: 24621470

[48] Coudriet GM He J Trucco M Mars WM Piganelli JD. Hepatocyte growth factor modulates interleukin-6 production in bone marrow derived macrophages: implications for inflammatory mediated diseases. PLoS One. (2010) 5:e15384. doi: 10.1371/journal.pone.0015384, PMID: 21072211 PMC2970559

[49] Rutella S Bonanno G Procoli A Mariotti A de Ritis DG Curti A. Hepatocyte growth factor favors monocyte differentiation into regulatory interleukin (IL)-10++IL-12low/neg accessory cells with dendritic-cell features. Blood. (2006) 108:218–27. doi: 10.1182/blood-2005-08-3141, PMID: 16527888

[50] Chandrasekher G Pothula S Maharaj G Bazan HE. Differential effects of hepatocyte growth factor and keratinocyte growth factor on corneal epithelial cell cycle protein expression, cell survival, and growth. Mol Vis. (2014) 20:24–37. PMID: 24426773 PMC3888494

[51] Sherr CJ McCormick F. The RB and p 53 pathways in cancer. Cancer Cell. (2002) 2:103–12. doi: 10.1016/s1535-6108(02)00102-2 12204530

[52] Elbasiony E Cho W Mittal SK Chauhan SK. Suppression of lipopolysaccharide-induced corneal opacity by hepatocyte growth factor. Sci Rep. (2022) 12:494. doi: 10.1038/s41598-021-04418-x, PMID: 35017561 PMC8752742

[53] Sharma GD He J Bazan HE. p38 and ERK1/2 coordinate cellular migration and proliferation in epithelial wound healing: evidence of cross-talk activation between MAP kinase cascades. J Biol Chem. (2003) 278:21989–97. doi: 10.1074/jbc.M302650200, PMID: 12663671

[54] He M Han T Wang Y Wu YH Qin WS du LZ. Effects of HGF and KGF gene silencing on vascular endothelial growth factor and its receptors in rat ultraviolet radiation-induced corneal neovascularization. Int J Mol Med. (2019) 43:1888–99. doi: 10.3892/ijmm.2019.4114, PMID: 30816491

[55] Matsumura A Kubota T Taiyoh H Fujiwara H Okamoto K Ichikawa D. HGF regulates VEGF expression via the c-met receptor downstream pathways, PI3K/Akt, MAPK and STAT3, in CT26 murine cells. Int J Oncol. (2013) 42:535–42. doi: 10.3892/ijo.2012.1728, PMID: 23233163

[56] Baxter RC. Signaling pathways of the insulin-like growth factor binding proteins. Endocr Rev. (2023) 44:753–78. doi: 10.1210/endrev/bnad008, PMID: 36974712 PMC10502586

[57] Stuard WL Titone R Robertson DM. The IGF/insulin-IGFBP axis in corneal development, wound healing, and disease. Front Endocrinol. (2020) 11:24. doi: 10.3389/fendo.2020.00024, PMID: 32194500 PMC7062709

[58] Trosan P Svobodova E Chudickova M Krulova M Zajicova A Holan V. The key role of insulin-like growth factor I in limbal stem cell differentiation and the corneal wound-healing process. Stem Cells Dev. (2012) 21:3341–50. doi: 10.1089/scd.2012.0180, PMID: 22873171 PMC3516427

[59] Slavin BR Sarhane KA von Guionneau N Hanwright PJ Qiu C Mao HQ. Insulin-like growth factor-1: a promising therapeutic target for peripheral nerve injury. Front Bioeng Biotechnol. (2021) 9:695850. doi: 10.3389/fbioe.2021.695850, PMID: 34249891 PMC8264584

[60] Yu F Gong D Yan D Wang H Witman N Lu Y. Enhanced adipose-derived stem cells with IGF-1-modified mRNA promote wound healing following corneal injury. Mol Ther. (2023) 31:2454–71. doi: 10.1016/j.ymthe.2023.05.002, PMID: 37165618 PMC10422019

[61] Nakamura M Chikama TI Nishida T. Characterization of insulin-like growth factor-1 receptors in rabbit corneal epithelial cells. Exp Eye Res. (2000) 70:199–204. doi: 10.1006/exer.1999.0775, PMID: 10655145

[62] Todorović V Peško P Micev M Bjelović M Budeč M Mićić M. Insulin-like growth factor-I in wound healing of rat skin. Regul Pept. (2008) 150:7–13. doi: 10.1016/j.regpep.2008.05.006, PMID: 18597865

[63] Ghiasi Z Gray T Tran P Dubielzig R Murphy C McCartney D. The effect of topical substance-P plus insulin-like growth factor-1 (IGF-1) on epithelial healing after photorefractive keratectomy in rabbits. Transl Vis Sci Technol. (2018) 7:12. doi: 10.1167/tvst.7.1.12, PMID: 29372114 PMC5782824

[64] Nagano T Nakamura M Nakata K Yamaguchi T Takase K Okahara A. Effects of substance P and IGF-1 in corneal epithelial barrier function and wound healing in a rat model of neurotrophic keratopathy. Invest Ophthalmol Vis Sci. (2003) 44:3810–5. doi: 10.1167/iovs.03-0189 12939296

[65] Patel R Zhu M Robertson DM. Shifting the IGF-axis: an age-related decline in human tear IGF-1 correlates with clinical signs of dry eye. Growth Hormon IGF Res. (2018) 40:69–73. doi: 10.1016/j.ghir.2018.02.001, PMID: 29452886 PMC5984117

[66] Wu YC Buckner BR Zhu M Cavanagh HD Robertson DM. Elevated IGFBP3 levels in diabetic tears: a negative regulator of IGF-1 signaling in the corneal epithelium. Ocul Surf. (2012) 10:100–7. doi: 10.1016/j.jtos.2012.01.004, PMID: 22482470 PMC3322367

[67] Jiang Y Ju Z Zhang J Liu X Tian J Mu G. Effects of insulin-like growth factor 2 and its receptor expressions on corneal repair. Int J Clin Exp Pathol. (2015) 8:10185–91. PMID: 26617727 PMC4637542

[68] Hassell JR Birk DE. The molecular basis of corneal transparency. Exp Eye Res. (2010) 91:326–35. doi: 10.1016/j.exer.2010.06.021, PMID: 20599432 PMC3726544

[69] Ferry RJ Jr Katz LE Grimberg A Cohen P Weinzimer SA. Cellular actions of insulin-like growth factor binding proteins. Horm Metab Res. (1999) 31:192–202. doi: 10.1055/s-2007-978719 10226802 PMC4151550

[70] Arnold DR Moshayedi P Schoen TJ Jones BE Chader GJ Waldbillig RJ. Distribution of IGF-I and -II, IGF binding proteins (IGFBPs) and IGFBP mRNA in ocular fluids and tissues: potential sites of synthesis of IGFBPs in aqueous and vitreous. Exp Eye Res. (1993) 56:555–65. doi: 10.1006/exer.1993.1069, PMID: 7684697

[71] Titone R Zhu M Robertson DM. Insulin mediates *de novo* nuclear accumulation of the IGF-1/insulin hybrid receptor in corneal epithelial cells. Sci Rep. (2018) 8:4378. doi: 10.1038/s41598-018-21031-7, PMID: 29531349 PMC5847585

[72] Conover CA Oxvig C. PAPP-A: a promising therapeutic target for healthy longevity. Aging Cell. (2017) 16:205–9. doi: 10.1111/acel.12564, PMID: 28035757 PMC5334524

[73] Argente J Chowen JA Pérez-Jurado LA Frystyk J Oxvig C. One level up: abnormal proteolytic regulation of IGF activity plays a role in human pathophysiology. EMBO Mol Med. (2017) 9:1338–45. doi: 10.15252/emmm.201707950, PMID: 28801361 PMC5623872

[74] Park SH Kim KW Kim JC. The role of insulin-like growth factor binding protein 2 (IGFBP2) in the regulation of corneal fibroblast differentiation. Invest Ophthalmol Vis Sci. (2015) 56:7293–302. doi: 10.1167/iovs.15-16616, PMID: 26559475

[75] Rao P Suvas PK Jerome AD Steinle JJ Suvas S. Role of insulin-like growth factor binding protein-3 in the pathogenesis of herpes stromal keratitis. Invest Ophthalmol Vis Sci. (2020) 61:46. doi: 10.1167/iovs.61.2.46, PMID: 32106295 PMC7329945

[76] Titone R Zhu M Robertson DM. Mutual regulation between IGF-1R and IGFBP-3 in human corneal epithelial cells. J Cell Physiol. (2019) 234:1426–41. doi: 10.1002/jcp.26948, PMID: 30078228 PMC6415676

[77] Lima MH Caricilli AM de Abreu LL Araújo EP Pelegrinelli FF Thirone ACP. Topical insulin accelerates wound healing in diabetes by enhancing the AKT and ERK pathways: a double-blind placebo-controlled clinical trial. PLoS One. (2012) 7:e36974. doi: 10.1371/journal.pone.0036974, PMID: 22662132 PMC3360697

[78] Wirostko B Rafii M Sullivan DA Morelli J Ding J. Novel therapy to treat corneal epithelial defects: a hypothesis with growth hormone. Ocul Surf. (2015) 13:204–212.e1. doi: 10.1016/j.jtos.2014.12.005, PMID: 26045234 PMC4498999

[79] Kolli S Bojic S Ghareeb AE Kurzawa-Akanbi M Figueiredo FC Lako M. The role of nerve growth factor in maintaining proliferative capacity, colony-forming efficiency, and the limbal stem cell phenotype. Stem Cells. (2019) 37:139–49. doi: 10.1002/stem.2921, PMID: 30599086 PMC6334532

[80] Huang EJ Reichardt LF. Trk receptors: roles in neuronal signal transduction. Annu Rev Biochem. (2003) 72:609–42. doi: 10.1146/annurev.biochem.72.121801.161629 12676795

[81] Lambiase A Manni L Bonini S Rama P Micera A Aloe L. Nerve growth factor promotes corneal healing: structural, biochemical, and molecular analyses of rat and human corneas. Invest Ophthalmol Vis Sci. (2000) 41:1063–9. PMID: 10752942

[82] Chen H Zhang J Dai Y Xu J. Nerve growth factor inhibits TLR3-induced inflammatory cascades in human corneal epithelial cells. J Inflamm. (2019) 16:27. doi: 10.1186/s12950-019-0232-0, PMID: 31889912 PMC6933932

[83] Micera A Lambiase A Puxeddu I Aloe L Stampachiacchiere B Levi-Schaffer F. Nerve growth factor effect on human primary fibroblastic-keratocytes: possible mechanism during corneal healing. Exp Eye Res. (2006) 83:747–57. doi: 10.1016/j.exer.2006.03.010, PMID: 16716299

[84] Blanco-Mezquita T Martinez-Garcia C Proença R Zieske JD Bonini S Lambiase A. Nerve growth factor promotes corneal epithelial migration by enhancing expression of matrix metalloprotease-9. Invest Ophthalmol Vis Sci. (2013) 54:3880–90. doi: 10.1167/iovs.12-10816, PMID: 23640040 PMC5110072

[85] Anitua E Muruzabal F Alcalde I Merayo-Lloves J Orive G. Plasma rich in growth factors (PRGF-endoret) stimulates corneal wound healing and reduces haze formation after PRK surgery. Exp Eye Res. (2013) 115:153–61. doi: 10.1016/j.exer.2013.07.007 23872360

[86] Muzi S Colafrancesco V Sornelli F Mantelli F Lambiase A Aloe L. Nerve growth factor in the developing and adult lacrimal glands of rat with and without inherited retinitis pigmentosa. Cornea. (2010) 29:1163–8. doi: 10.1097/ICO.0b013e3181d3d3f9, PMID: 20595895

[87] Aloe L Tirassa P Lambiase A. The topical application of nerve growth factor as a pharmacological tool for human corneal and skin ulcers. Pharmacol Res. (2008) 57:253–8. doi: 10.1016/j.phrs.2008.01.010, PMID: 18329283

[88] Aloe L Rocco ML Balzamino BO Micera A. Nerve growth factor: a focus on neuroscience and therapy. Curr Neuropharmacol. (2015) 13:294–303. doi: 10.2174/1570159x13666150403231920, PMID: 26411962 PMC4812798

[89] Wu D Qian T Hong J Li G Shi W Xu J. Micro RNA-494 inhibits nerve growth factor-induced cell proliferation by targeting cyclin D1 in human corneal epithelial cells. Mol Med Rep. (2017) 16:4133–42. doi: 10.3892/mmr.2017.7083, PMID: 28765880

[90] Sun Z Hu W Yin S Lu X Zuo W Ge S. NGF protects against oxygen and glucose deprivation-induced oxidative stress and apoptosis by up-regulation of HO-1 through MEK/ERK pathway. Neurosci Lett. (2017) 641:8–14. doi: 10.1016/j.neulet.2017.01.046, PMID: 28115238

[91] Park JH Kang SS Kim JY Tchah H. Nerve growth factor attenuates apoptosis and inflammation in the diabetic cornea. Invest Ophthalmol Vis Sci. (2016) 57:6767–75. doi: 10.1167/iovs.16-19747, PMID: 27978558

[92] Ding Z Jiang M Qian J Gu D Bai H Cai M. Role of transforming growth factor-β in peripheral nerve regeneration. Neural Regen Res. (2024) 19:380–6. doi: 10.4103/1673-5374.377588 37488894 PMC10503632

[93] Nishida K Kinoshita S Yokoi N Kaneda M Hashimoto K Yamamoto S. Immunohistochemical localization of transforming growth factor-beta 1, -beta 2, and -beta 3 latency-associated peptide in human cornea. Invest Ophthalmol Vis Sci. (1994) 35:3289–94. PMID: 8045718

[94] Singh V Jaini R Torricelli AA Santhiago MR Singh N Ambati BK. TGFβ and PDGF-B signaling blockade inhibits myofibroblast development from both bone marrow-derived and keratocyte-derived precursor cells in vivo. Exp Eye Res. (2014) 121:35–40. doi: 10.1016/j.exer.2014.02.013, PMID: 24582892 PMC3996053

[95] Bhowmick NA Zent R Ghiassi M McDonnell M Moses HL. Integrin beta 1 signaling is necessary for transforming growth factor-beta activation of p38MAPK and epithelial plasticity. J Biol Chem. (2001) 276:46707–13. doi: 10.1074/jbc.M106176200, PMID: 11590169

[96] Terai K Call MK Liu H Saika S Liu CY Hayashi Y. Crosstalk between TGF-beta and MAPK signaling during corneal wound healing. Invest Ophthalmol Vis Sci. (2011) 52:8208–15. doi: 10.1167/iovs.11-8017, PMID: 21917935 PMC3208026

[97] Er H Uzmez E. Effects of transforming growth factor-beta 2, interleukin 6 and fibronectin on corneal epithelial wound healing. Eur J Ophthalmol. (1998) 8:224–9. doi: 10.1177/112067219800800404, PMID: 9891893

[98] Wang YC Zolnik OB Yasuda S Yeh LK Yuan Y Kao W. Transforming growth factor beta receptor 2 (Tgfbr 2) deficiency in keratocytes results in corneal ectasia. Ocul Surf. (2023) 29:557–65. doi: 10.1016/j.jtos.2023.06.014, PMID: 37393064

[99] Bettahi I Sun H Gao N Wang F Mi X Chen W. Genome-wide transcriptional analysis of differentially expressed genes in diabetic, healing corneal epithelial cells: hyperglycemia-suppressed TGFβ3 expression contributes to the delay of epithelial wound healing in diabetic corneas. Diabetes. (2014) 63:715–27. doi: 10.2337/db13-1260, PMID: 24306208 PMC3900551

[100] Rocher M Robert PY Desmoulière A. The myofibroblast, biological activities and roles in eye repair and fibrosis. A focus on healing mechanisms in avascular cornea. Eye. (2020) 34:232–40. doi: 10.1038/s41433-019-0684-8, PMID: 31767967 PMC7002667

[101] Li ZY Chen ZL Zhang T Wei C Shi WY. TGF-β and NF-κB signaling pathway crosstalk potentiates corneal epithelial senescence through an RNA stress response. Aging. (2016) 8:2337–54. doi: 10.18632/aging.101050, PMID: 27713146 PMC5115892

[102] Benito MJ Calder V Corrales RM García-Vázquez C Narayanan S Herreras JM. Effect of TGF-β on ocular surface epithelial cells. Exp Eye Res. (2013) 107:88–100. doi: 10.1016/j.exer.2012.11.017 23220729

[103] Fink MK Giuliano EA Tandon A Mohan RR. Therapeutic potential of pirfenidone for treating equine corneal scarring. Vet Ophthalmol. (2015) 18:242–50. doi: 10.1111/vop.12194, PMID: 25041235 PMC4295017

[104] Saikia P Thangavadivel S Medeiros CS Lassance L de Oliveira RC Wilson SE. IL-1 and TGF-β modulation of epithelial basement membrane components perlecan and nidogen production by corneal stromal cells. Invest Ophthalmol Vis Sci. (2018) 59:5589–98. doi: 10.1167/iovs.18-25202, PMID: 30480706 PMC6262649

[105] Heldin CH Westermark B. Platelet-derived growth factor: mechanism of action and possible *in vivo* function. Cell Regul. (1990) 1:555–66. doi: 10.1091/mbc.1.8.555, PMID: 1964089 PMC361590

[106] Daniels JT Khaw PT. Temporal stimulation of corneal fibroblast wound healing activity by differentiating epithelium *in vitro*. Invest Ophthalmol Vis Sci. (2000) 41:3754–62. PMID: 11053273

[107] Nishida T. Translational research in corneal epithelial wound healing. Eye Contact Lens. (2010) 36:300–4. doi: 10.1097/ICL.0b013e3181f016d0 20724848

[108] Kamiyama K Iguchi I Wang X Imanishi J. Effects of PDGF on the migration of rabbit corneal fibroblasts and epithelial cells. Cornea. (1998) 17:315–25. doi: 10.1097/00003226-199805000-00013 9603389

[109] Hoppenreijs VP Pels E Vrensen GF Felten PC Treffers WF. Platelet-derived growth factor: receptor expression in corneas and effects on corneal cells. Invest Ophthalmol Vis Sci. (1993) 34:637–49. PMID: 8449682

[110] Williamson JR Monck JR. Hormone effects on cellular Ca^2+^ fluxes. Annu Rev Physiol. (1989) 51:107–24. doi: 10.1146/annurev.ph.51.030189.000543 2496641

[111] Denk PO Knorr M. The in vitro effect of platelet-derived growth factor isoforms on the proliferation of bovine corneal stromal fibroblasts depends on cell density. Graefes Arch Clin Exp Ophthalmol. (1997) 235:530–4. doi: 10.1007/BF00947012, PMID: 9285224

[112] Schultz T Conrad-Hengerer I Hengerer FH Dick HB. Intraocular pressure variation during femtosecond laser-assisted cataract surgery using a fluid-filled interface. J Cataract Refract Surg. (2013) 39:22–7. doi: 10.1016/j.jcrs.2012.10.038, PMID: 23245360

[113] Wei J Tang H Xu ZQ Li B Xie LQ Xu GX. Expression and function of PDGF-α in columnar epithelial cells of age-related cataracts patients. Genet Mol Res. (2015) 14:13320–7. doi: 10.4238/2015.October.26.28, PMID: 26535645

[114] Jin L Yang R Geng L Xu A. Fibroblast growth factor-based pharmacotherapies for the treatment of obesity-related metabolic complications. Annu Rev Pharmacol Toxicol. (2023) 63:359–82. doi: 10.1146/annurev-pharmtox-032322-093904 36100222

[115] Shin E Lim DH Han J Nam DH Park K Ahn MJ. Markedly increased ocular side effect causing severe vision deterioration after chemotherapy using new or investigational epidermal or fibroblast growth factor receptor inhibitors. BMC Ophthalmol. (2020) 20:19. doi: 10.1186/s12886-019-1285-9, PMID: 31918686 PMC6953164

[116] Cai J Zhou Q Wang Z Guo R Yang R Yang X. Comparative analysis of KGF-2 and bFGF in prevention of excessive wound healing and scar formation in a corneal alkali burn model. Cornea. (2019) 38:1430–7. doi: 10.1097/ICO.0000000000002134, PMID: 31490279

[117] Reneker LW Irlmeier RT Shui YB Liu Y Huang AJW. Histopathology and selective biomarker expression in human meibomian glands. Br J Ophthalmol. (2020) 104:999–1004. doi: 10.1136/bjophthalmol-2019-314466, PMID: 31585964 PMC7361036

[118] Zhang J Upadhya D Lu L Reneker LW. Fibroblast growth factor receptor 2 (FGFR2) is required for corneal epithelial cell proliferation and differentiation during embryonic development. PLoS One. (2015) 10:e0117089. doi: 10.1371/journal.pone.0117089, PMID: 25615698 PMC4304804

[119] Joseph R Boateng A Srivastava OP Pfister RR. Role of fibroblast growth factor receptor 2 (FGFR2) in corneal stromal thinning. Invest Ophthalmol Vis Sci. (2023) 64:40. doi: 10.1167/iovs.64.12.40, PMID: 37750740 PMC10541240

[120] Qu X Carbe C Tao C Powers A Lawrence R van Kuppevelt TH. Lacrimal gland development and Fgf 10-Fgfr2b signaling are controlled by 2-O- and 6-O-sulfated heparan sulfate. J Biol Chem. (2011) 286:14435–44. doi: 10.1074/jbc.M111.225003, PMID: 21357686 PMC3077643

[121] Tsau C Ito M Gromova A Hoffman MP Meech R Makarenkova HP. Barx 2 and Fgf 10 regulate ocular glands branching morphogenesis by controlling extracellular matrix remodeling. Development. (2011) 138:3307–17. doi: 10.1242/dev.066241, PMID: 21750040 PMC3133920

[122] Ma M Zhang Z Niu W Zheng W Kelimu J Ke B. Fibroblast growth factor 10 upregulates the expression of mucins in rat conjunctival epithelial cells. Mol Vis. (2011) 17:2789–97. PMID: 22065934 PMC3209430

[123] Tao H Shimizu M Kusumoto R Ono K Noji S Ohuchi H. A dual role of FGF10 in proliferation and coordinated migration of epithelial leading edge cells during mouse eyelid development. Development. (2005) 132:3217–30. doi: 10.1242/dev.01892 15958512

[124] Thotakura S Basova L Makarenkova HP. FGF gradient controls boundary position between proliferating and differentiating cells and regulates lacrimal gland growth dynamics. Front Genet. (2019) 10:362. doi: 10.3389/fgene.2019.00362, PMID: 31191595 PMC6546953

[125] Li J Zhang R Wang C Wang X Xu M Ma J. Activation of the small GTPase rap 1 inhibits choroidal neovascularization by regulating cell junctions and ROS generation in rats. Curr Eye Res. (2018) 43:934–40. doi: 10.1080/02713683.2018.1454477, PMID: 29601231

[126] Li L Wang H Pang S Wang L Fan Z Ma C. Rh FGF-21 accelerates corneal epithelial wound healing through the attenuation of oxidative stress and inflammatory mediators in diabetic mice. J Biol Chem. (2023) 299:105127. doi: 10.1016/j.jbc.2023.105127, PMID: 37544647 PMC10481360

[127] Wilson SE Li Q Mohan RR Tervo T Vesaluoma M Bennett GL. Lacrimal gland growth factors and receptors: lacrimal fibroblastic cells are a source of tear HGF. Adv Exp Med Biol. (1998) 438:625–8. doi: 10.1007/978-1-4615-5359-5_88, PMID: 9634946

[128] Teranishi S Kimura K Kawamoto K Nishida T. Protection of human corneal epithelial cells from hypoxia-induced disruption of barrier function by keratinocyte growth factor. Invest Ophthalmol Vis Sci. (2008) 49:2432–7. doi: 10.1167/iovs.07-1464, PMID: 18362114

[129] Wilson SE Walker JW Chwang EL He YG. Hepatocyte growth factor, keratinocyte growth factor, their receptors, fibroblast growth factor receptor-2, and the cells of the cornea. Invest Ophthalmol Vis Sci. (1993) 34:2544–61. PMID: 8392040

[130] Kareem ZY McLaughlin PJ Kumari R. Opioid growth factor receptor: anatomical distribution and receptor colocalization in neurons of the adult mouse brain. Neuropeptides. (2023) 99:102325. doi: 10.1016/j.npep.2023.102325, PMID: 36812665

[131] Cheng F McLaughlin PJ Verderame MF Zagon IS. The OGF-OGFr axis utilizes the p16INK4a and p21WAF1/CIP1 pathways to restrict normal cell proliferation. Mol Biol Cell. (2009) 20:319–27. doi: 10.1091/mbc.e08-07-0681, PMID: 18923142 PMC2613082

[132] McLaughlin PJ Sassani JW Titunick MB Zagon IS. Efficacy and safety of a novel naltrexone treatment for dry eye in type 1 diabetes. BMC Ophthalmol. (2019) 19:35. doi: 10.1186/s12886-019-1044-y, PMID: 30691415 PMC6348650

[133] McLaughlin PJ Sassani JW Diaz D Zagon IS. Elevated opioid growth factor alters the limbus in type 1 diabetic rats. J Diabetes Clin Res. (2023) 5:1–10. doi: 10.33696/diabetes.4.054, PMID: 37304310 PMC10254076

[134] Zagon IS Sassani JW Purushothaman I McLaughlin PJ. Blockade of OGFr delays the onset and reduces the severity of diabetic ocular surface complications. Exp Biol Med. (2021) 246:629–36. doi: 10.1177/1535370220972060, PMID: 33203224 PMC7934152

[135] Immonen JA Zagon IS McLaughlin PJ. Selective blockade of the OGF-OGFr pathway by naltrexone accelerates fibroblast proliferation and wound healing. Exp Biol Med. (2014) 239:1300–9. doi: 10.1177/1535370214543061, PMID: 25030485

[136] Zagon IS Sassani JW Purushothaman I McLaughlin PJ. Dysregulation of the OGF-OGFr pathway correlates with elevated serum OGF and ocular surface complications in the diabetic rat. Exp Biol Med. (2020) 245:1414–21. doi: 10.1177/1535370220940273, PMID: 32640891 PMC7441350

[137] Zagon IS Klocek MS Sassani JW McLaughlin PJ. Dry eye reversal and corneal sensation restoration with topical naltrexone in diabetes mellitus. Arch Ophthalmol. (2009) 127:1468–73. doi: 10.1001/archophthalmol.2009.270, PMID: 19901212 PMC2840396

[138] Klocek MS Sassani JW McLaughlin PJ Zagon IS. Naltrexone and insulin are independently effective but not additive in accelerating corneal epithelial healing in type I diabetic rats. Exp Eye Res. (2009) 89:686–92. doi: 10.1016/j.exer.2009.06.010, PMID: 19576213 PMC2757498

[139] Bussolino F di Renzo MF Ziche M Bocchietto E Olivero M Naldini L. Hepatocyte growth factor is a potent angiogenic factor which stimulates endothelial cell motility and growth. J Cell Biol. (1992) 119:629–41. doi: 10.1083/jcb.119.3.629, PMID: 1383237 PMC2289675

[140] Beheshtizadeh N Gharibshahian M Bayati M Maleki R Strachan H Doughty S. Vascular endothelial growth factor (VEGF) delivery approaches in regenerative medicine. Biomed Pharmacother. (2023) 166:115301. doi: 10.1016/j.biopha.2023.115301, PMID: 37562236

[141] Giannaccare G Pellegrini M Bernabei F Scorcia V Campos E. Ocular surface system alterations in ocular graft-versus-host disease: all the pieces of the complex puzzle. Graefes Arch Clin Exp Ophthalmol. (2019) 257:1341–51. doi: 10.1007/s00417-019-04301-6, PMID: 30944986

[142] Amano S Rohan R Kuroki M Tolentino M Adamis AP. Requirement for vascular endothelial growth factor in wound- and inflammation-related corneal neovascularization. Invest Ophthalmol Vis Sci. (1998) 39:18–22. PMID: 9430540

[143] Giannaccare G Pellegrini M Bovone C Spena R Senni C Scorcia V. Anti-VEGF treatment in corneal diseases. Curr Drug Targets. (2020) 21:1159–80. doi: 10.2174/1389450121666200319111710 32189591

[144] Goldhardt R Batawi HIM Rosenblatt M Lollett IV Park JJ Galor A. Effect of anti-vascular endothelial growth factor therapy on corneal nerves. Cornea. (2019) 38:559–64. doi: 10.1097/ICO.0000000000001871, PMID: 30933961

[145] Pan Z Fukuoka S Karagianni N Guaiquil VH Rosenblatt MI. Vascular endothelial growth factor promotes anatomical and functional recovery of injured peripheral nerves in the avascular cornea. FASEB J. (2013) 27:2756–67. doi: 10.1096/fj.12-225185, PMID: 23568776 PMC3688738

[146] Jin J Guan M Sima J Gao G Zhang M Liu Z. Decreased pigment epithelium-derived factor and increased vascular endothelial growth factor levels in pterygia. Cornea. (2003) 22:473–7. doi: 10.1097/00003226-200307000-00015, PMID: 12827055

[147] Yeh SI Yu SH Chu HS Huang CT Tsao YP Cheng CM. Pigment epithelium-derived factor peptide promotes corneal nerve regeneration: an *in vivo* and *in vitro* study. Invest Ophthalmol Vis Sci. (2021) 62:23. doi: 10.1167/iovs.62.1.23, PMID: 33481984 PMC7838554

[148] Ho TC Chen SL Wu JY Ho MY Chen LJ Hsieh JW. PEDF promotes self-renewal of limbal stem cell and accelerates corneal epithelial wound healing. Stem Cells. (2013) 31:1775–84. doi: 10.1002/stem.1393, PMID: 23553951

[149] Versura P Giannaccare G Pellegrini M Sebastiani S Campos EC. Neurotrophic keratitis: current challenges and future prospects. Eye Brain. (2018) 10:37–45. doi: 10.2147/EB.S117261, PMID: 29988739 PMC6029608

[150] Hao M Cheng Y Wu J Cheng Y Wang J. Clinical observation of recombinant human nerve growth factor in the treatment of neurotrophic keratitis. Int J Ophthalmol. (2023) 16:60–6. doi: 10.18240/ijo.2023.01.09, PMID: 36659958 PMC9815986

[151] Sridhar MS. Anatomy of cornea and ocular surface. Indian J Ophthalmol. (2018) 66:190–4. doi: 10.4103/ijo.IJO_646_17, PMID: 29380756 PMC5819093

[152] Sun X Song W Teng L Huang Y Liu J Peng Y. MiRNA 24-3p-rich exosomes functionalized DEGMA-modified hyaluronic acid hydrogels for corneal epithelial healing. Bioact Mater. (2022) 25:640–56. doi: 10.1016/j.bioactmat.2022.07.011, PMID: 37056274 PMC10086767

